# Integrating Multimodal Neuroimaging of Error Monitoring to Estimate Future Anxiety in Adolescents

**DOI:** 10.1001/jamanetworkopen.2025.39133

**Published:** 2025-10-23

**Authors:** Emilio Alejandro Valadez, Stefania Conte, John E. Richards, Yi Feng, Lucrezia Liuzzi, Marco McSweeney, Enda Tan, George A. Buzzell, Santiago Morales, Anderson M. Winkler, Elise M. Cardinale, Lauren K. White, Daniel S. Pine, Nathan A. Fox

**Affiliations:** 1 Department of Psychology, University of Southern California, Los Angeles; 2 Department of Psychology, Binghamton University, Binghamton, New York; 3 Department of Psychology, University of South Carolina, Columbia; 4 Department of Psychology, University of California, Los Angeles; 5 Emotion and Development Branch, National Institute of Mental Health, Bethesda, Maryland; 6 Department of Human Development and Quantitative Methodology, University of Maryland, College Park; 7 Department of Psychology, University of British Columbia, Vancouver, British Columbia, Canada; 8 Department of Psychology and Center for Children and Families, Florida International University, University Park; 9 Department of Human Genetics, University of Texas Rio Grande Valley, Brownsville; 10 Department of Psychology, The Catholic University of America, Washington, DC; 11 Lifespan Brain Institute of the Children’s Hospital of Philadelphia and Penn Medicine, Philadelphia, Pennsylvania

## Abstract

**Importance:**

Anxiety disorders are highly prevalent and associated with heightened error monitoring, the detection of one’s mistakes. However, error monitoring, anxiety, and their associations change throughout adolescence, limiting the ability to estimate future anxiety trajectories during this period.

**Objective:**

To ascertain whether measures of error monitoring obtained via the integration of electroencephalogram (EEG) and functional magnetic resonance imaging (fMRI) improve estimations of future anxiety compared with EEG or fMRI alone, in adolescents with or without a history of behaviorally inhibited temperament.

**Design, Setting, and Participants:**

This longitudinal cohort study was conducted at a university research laboratory and government research hospital. Study assessments took place between January 2014 and July 2019, and data analyses were completed in January 2025. A community sample of infants completed a laboratory screening at age 4 months. A subset of these infants was oversampled to maximize variability of early temperament and followed up throughout adolescence. At ages 13 and 15 years, participants completed a flanker task during an EEG session and separate fMRI session. Brain activity at age 13 years and its change from 13 to 15 years of age were evaluated as potential risk factors for anxiety.

**Main Outcomes and Measures:**

Change in anxiety from age 13 to 15 years as measured using clinical interviews and questionnaires. Hypotheses were formulated after data collection.

**Results:**

Analyses included 176 adolescents with neuroimaging data (92 females at birth [52.3%]; 5 Asian individuals [2.8%], 21 Black or African American individuals [11.9%], 11 Hispanic or Latino individuals [6.3%], 133 White individuals [75.6%], and 6 individuals of other [3.4%] race and ethnicity). Among neural variables (EEG-only, fMRI-only, and EEG-fMRI fusion), only the EEG-fMRI fusion scores explained additional variance in anxiety change scores (change in *R*^2^ = 0.25; *P* = .001) beyond demographics (sex, racial and ethnic minority status) and anxiety at age 13 years. Planned follow-up analyses revealed that early temperament interacted with dorsal anterior cingulate activity at age 13 years (*β* = 0.40; *B* = 8.77; 95% CI, 0.74-16.79; *P* = .03) and with changes in posterior cingulate activity (*β* = −0.42; *B* = −16.89; 95% CI, –28.21 to –5.57; *P* = .003).

**Conclusions and Relevance:**

This cohort study found that integrating multimodal neuroimaging measures of error monitoring was associated with improved estimations of future anxiety in youths over and above each modality separately. Early temperament interacted with error monitoring to further differentiate anxiety trajectories; yet, these interactions differed across brain regions, highlighting the value of incorporating the complementary temporal and spatial information of EEG and fMRI.

## Key Points

**Question:**

Does integrating electroencephalogram (EEG)-based and functional magnetic resonance imaging (fMRI)-based measures of error monitoring improve estimation of future anxiety changes during adolescence?

**Findings:**

In this cohort study of 176 adolescents, only error monitoring scores derived from a novel EEG-fMRI fusion approach were associated with improved estimation of future anxiety changes. The EEG-fMRI fusion scores explained additional variance in later anxiety scores over and above demographic information, baseline anxiety, EEG-only measures, and fMRI-only measures.

**Meaning:**

Findings of this study suggest that integrating the complementary temporal and spatial precision of EEG and fMRI can improve estimation of adolescents’ future mental health trajectories.

## Teaser

This cohort study evaluates whether measures of error monitoring with electroencephalogram and functional magnetic resonance imaging (fMRI) vs fMRI alone are associated with improved estimates of future anxiety in adolescents with or without a history of behavioral inhibition.

## Introduction

Anxiety disorders are the most prevalent form of psychopathology in the US.^1^ One of the strongest and earliest known risk factors of anxiety disorders is behavioral inhibition (BI), a temperament involving distress to novelty.^2^ Toddlers with BI face 3 to 6 times greater risk for an adolescent anxiety disorder than those with other temperament styles.^3^ Nevertheless, only about 40% to 60% of children with BI develop an anxiety disorder,^3,4^ underscoring the importance of moderators.

Cognitive control, the goal-oriented monitoring and adjustment of behavior, differentiates anxiety outcomes among children with a history of BI.^5,6,7,8,9^ Relative to nonanxious children with BI, children with both high anxiety and BI demonstrate greater cognitive control skills^6,7^—specifically, heightened reactive control skills requiring in-the-moment processing (as opposed to proactive, future-oriented control skills)^10,11,12,13,14,15^ and heightened detection of conflict and potential threats, such as error monitoring.^15,16,17^

Error monitoring enables detection of mistakes.^18,19^ Error-related negativity (ERN) is a frontocentral negative-going voltage deflection measured via electroencephalogram (EEG) that is larger (indicating greater polarization relative to pretrial baseline amplitude) following errors than correct button responses.^18,19^ A larger ERN relates to anxiety in young children and adults.^20^ In pivotal prospective studies, a larger ERN was associated with worsening anxiety when controlling for baseline anxiety, both in a relatively large community sample of 6-year-old children^21^ and in a smaller clinically anxious sample of 8- to 14-year-old girls.^22^ Thus, the ERN may be an anxiety vulnerability biomarker.^20^

Age-related changes manifest in ERN amplitude,^20,23,24^ anxiety,^25^ and their association,^20,26,27,28^ limiting conclusions on developmental pathophysiology. Adolescence encapsulates an inflection point both for anxiety risk^25^ and ERN amplitude,^23^ creating a particular need for research during this period. Although the ERN’s main neural generators are the dorsal anterior cingulate cortex (dACC) and posterior cingulate cortex (PCC),^24,26,29,30,31^ age-related shifts occur in ERN-related brain activity, with the PCC’s contribution to the ERN increasing across adolescence in youths with or without anxiety.^24,29^ The dACC supports error or conflict detection^32,33,34^ and threat orienting.^35,36,37^ The PCC, central to the default mode network and connected to the prefrontal cortex, may facilitate flexible attentional reshifting as children mature.^38^ Therefore, whereas the dACC develops earlier and may support orienting attention toward errors and other threats (increasing anxiety risk), the PCC may support adaptive reshifting of attention as children mature (decreasing anxiety risk).^26^ Together, the literature suggests that individual differences in the development of dACC and PCC reactivity to errors may be associated with anxiety symptoms, especially among but not limited to youths with a history of BI. Improved separation of these regions via more precise ERN measurement may improve understanding of anxiety trajectories among adolescents.

The present study addresses this issue by applying a novel multimodal imaging approach to a longitudinal sample of adolescents recruited during infancy. Specifically, we aimed to ascertain whether measures of error monitoring obtained via the integration of EEG and functional magnetic resonance imaging (fMRI) improve estimations of future anxiety compared with EEG or fMRI alone. EEG and fMRI were combined (EEG-fMRI fusion) to capitalize on their complementary strengths to improve ERN measurement precision. It was hypothesized that EEG-fMRI fusion measures of error monitoring would explain additional variance in anxiety trajectories (change in anxiety across age 13 through 15 years) beyond EEG or fMRI alone. It was further hypothesized that among youths with a history of BI, greater ERN-related brain activity in the dACC would be associated with worsening anxiety, whereas activity in the PCC would show the opposite pattern.

## Methods

This longitudinal cohort study was conducted in a university research laboratory and government research hospital. Study assessments occurred between January 2014 and July 2019, and data analyses were completed in January 2025. Written informed consent and assent were obtained from parents and participants, respectively, at each assessment. The study and each visit protocol were approved by the institutional review boards of the National Institute of Mental Health Intramural Research Program (fMRI laboratory visits) and the University of Maryland, College Park (all other data collection). We followed the Strengthening the Reporting of Observational Studies in Epidemiology (STROBE) reporting guideline.^39^

### Participants

A community sample of infants completed temperament screening^40,41^ at age 4 months. A subset of these infants oversampled for high-motor and either high-positive or high-negative reactivity was enrolled, yielding a sample with enhanced variability of temperamental reactivity. This subset continued to participate through adolescence. Participants’ race and ethnicity were identified by parents at the time of infant recruitment using the following categories: Asian, Black or African American, Hispanic or Latino, White, and other (no further information available). Race and ethnicity data were collected to enable reporting of demographic characteristics and to assess the generalizability of study findings.

### EEG-fMRI Fusion Measures

**Figure 1.**
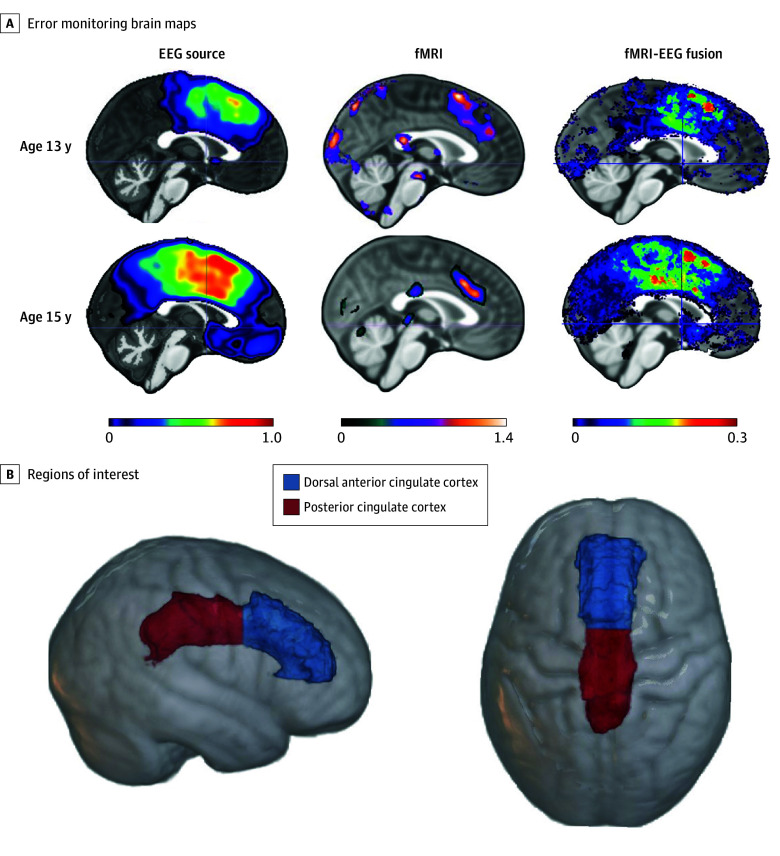
Neural Measures of Error Monitoring Figure adapted with permission from Conte et al.^24^ EEG indicates electroencephalogram; fMRI, functional magnetic resonance imaging.

At ages 13 and 15 years, participants were administered a modified flanker task^3^ during separate EEG and fMRI acquisitions; flanker task details are provided in the eMethods in Supplement 1. The EEG and MRI acquisition and analytic procedures for these data have been reported elsewhere^24^ and are included in the eMethods in Supplement 1. These procedures were performed separately at each time point. Briefly, EEG was recorded using a 128-channel geodesic sensor net, and structural and fMRI were acquired in a 3-Tesla scanner (Discovery MR750; GE HealthCare). Cortical source analysis was applied to the EEG data, focusing on the time point at which the participant’s ERN difference between incongruent-error and incongruent-correct trials was maximal. The cortical source analysis used structural MRI to generate participant-specific brain maps consisting of a tetrahedral mesh with current density reconstruction (CDR) values. Each CDR value indicates the degree to which that location contributed to the ERN. CDR maps from incongruent-correct trials were subtracted from those of incongruent-error trials to generate CDR difference maps reflecting activity unique to errors (Figure 1A, EEG source). Separately, fMRI analyses focused on a contrast reflecting incongruent-error minus incongruent-correct from the mean of 4 runs of the flanker task, which were registered to the participant’s T1 space (3-mm voxels, no interpolation) (Figure 1A, fMRI).

To integrate the fMRI and EEG data, the fMRI results were used to condition the EEG source results. Any fMRI voxel that overlapped with an element of the CDR-difference tetrahedral mesh was multiplied by that CDR-difference value. Note that not all tetrahedral elements intersected with the fMRI voxels and vice versa due to resolution differences between the fMRI data and EEG source models. The values resulting from this multiplication are referred to as EEG-fMRI fusion scores (Figure 1A, fMRI-EEG fusion).

### Statistical Analysis

**Figure 2.**
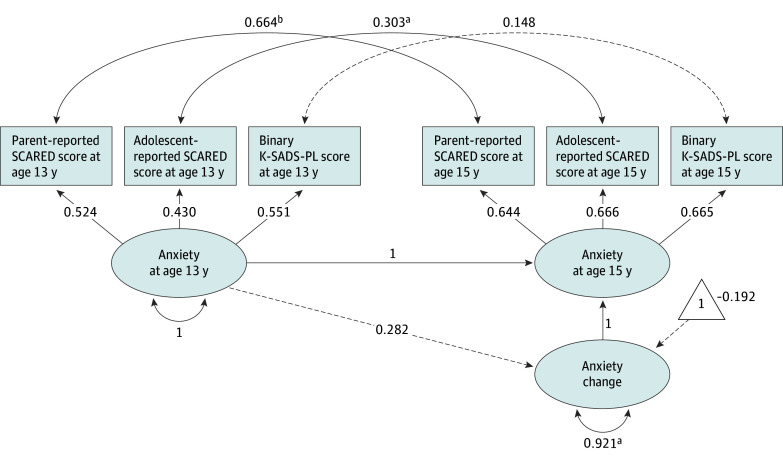
Latent Change Score Model for Anxiety Standardized anxiety change estimates are shown. Unstandardized factor loadings were constrained to be equal across time for measurement invariance. Robust root mean square error of approximation = 0.030, standardized root mean squared residual = 0.049, and robust comparative fit index = 0.995. Solid lines represent statistically significant paths (*P* < .05), dashed lines represent nonsignificant paths (*P* > .05), and 1 represents the latent change score's intercept (ie, mean). SCARED indicates Screen for Child Anxiety Related Emotional Disorders (score range: 0-82, with the higher scores indicating greater anxiety); K-SADS-PL, Schedule for Affective Disorders and Schizophrenia for School-Age Children Present and Lifetime Version (which provides diagnostic information but not scores). ^a^*P* < .05. ^b^*P* < .001.

For bivariate correlations involving all key study measures, see eFigure 1 in Supplement 1. A latent change score (LCS) model estimated anxiety change from age 13 to 15 years (Figure 2). At each age, anxiety was measured as a latent factor with 3 indicators: parent-reported total score on the Screen for Child Anxiety Related Emotional Disorders (SCARED; score range: 0-82, with the higher scores indicating greater anxiety),^42^ adolescent-reported SCARED total score, and a binary score indicating whether the participant currently met criteria for any anxiety diagnosis based on the clinical interview using the Schedule for Affective Disorders and Schizophrenia for School-Age Children Present and Lifetime Version (K-SADS-PL, which provides diagnostic information but not scores).^43^ Details about anxiety and temperament measures are available in the eMethods in Supplement 1. For inclusion in LCS models, means of neural data from each modality (EEG, fMRI, and EEG-fMRI fusion) and time point (13 and 15 years) were calculated within 2 a priori regions of interest (dACC and PCC) (Figure 1B). Region of interest–specific LCS models estimated change in dACC and PCC reactivity to errors separately for each modality, yielding 6 neural LCS models (eFigure 2 in Supplement 1).

**Figure 3.**
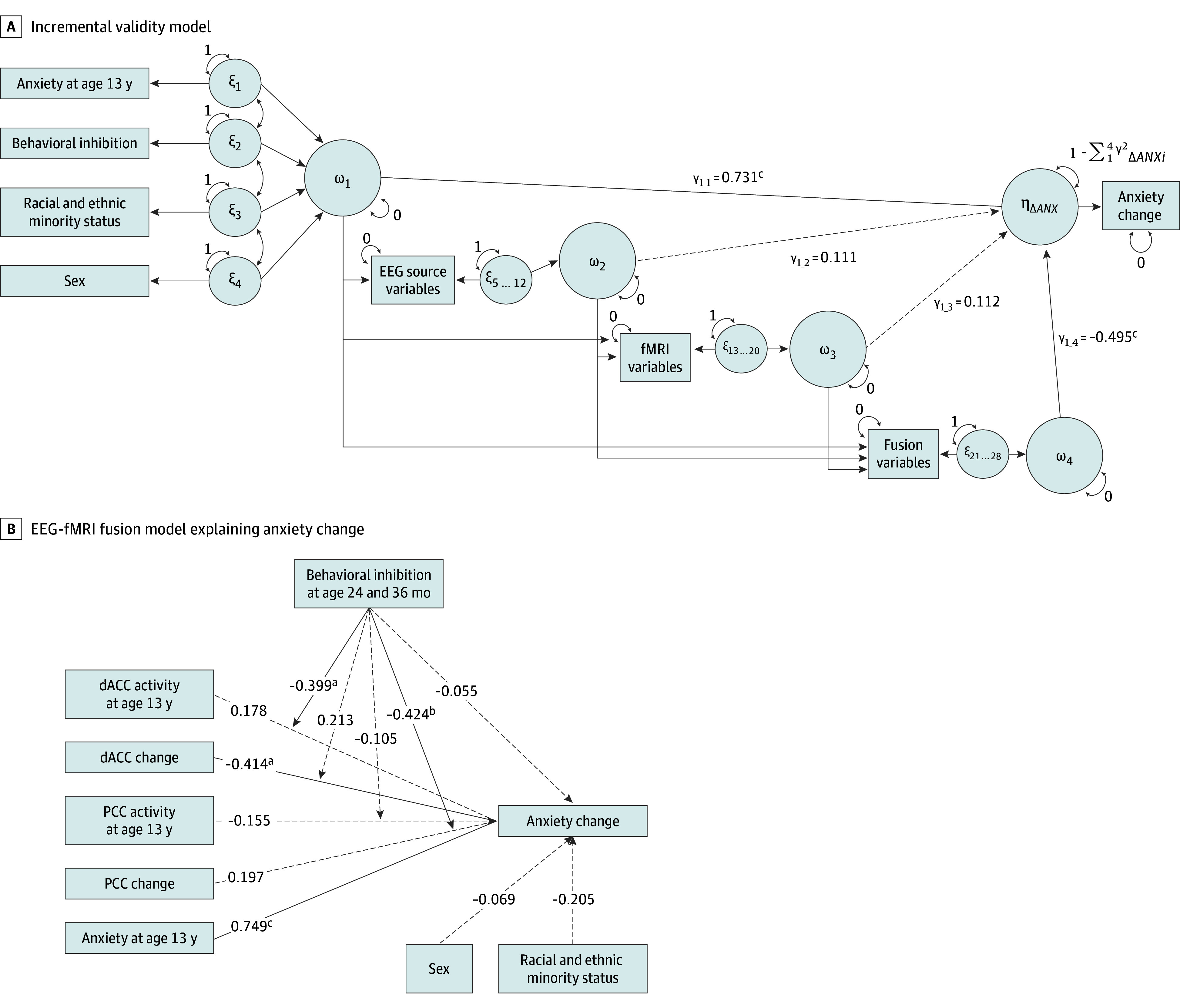
Model Results For the complete incremental validity model, see eFigure 3 in Supplement 1. Path coefficients are standardized. Arrows represent statistically significant paths (*P* < .05), and dashed arrows represent nonsignificant paths (*P* > .05). Fit indices are not reported because models are just-identified. Due to small cell sizes for racial and ethnic minority status, non-Hispanic White was coded as 0 and all other groups were coded as 1. EEG indicates electroencephalogram; fMRI, functional magnetic resonance imaging; dACC, dorsal anterior cingulate cortex; and PCC, posterior cingulate cortex. ε represents exogenous phantom variables (one for each observed variable); ω, block-level latent factors (one for each block of factors), γ, path coefficients representing the multiple correlations between each block and the outcome variable while removing effects of previous blocks; and ηΔ*ANX*, latent factor for anxiety change. ^a^*P* < .05. ^b^*P* < .01. ^c^*P* < .001.

Next, to assess the extent to which each modality incrementally explained anxiety change, we extracted the estimated change scores from each LCS model and computed interaction terms with BI, and these factors were included in a path model estimating the change in anxiety from age 13 to 15 years. Figure 3A shows a simplified model diagram; the complete model is illustrated in eFigure 3 in Supplement 1. This path model and subsequent analyses were fit separately from the LCS models to reduce model complexity and improve interpretability of results. The combined path model was parameterized based on recommendations by Feng and Hancock^44^; specifically, factors were entered as 4 separate blocks, with strategically parameterized path coefficients, thus allowing simultaneous estimation and statistical testing of the sequential incremental changes in *R*^2^ with the addition of each block. In this path model, block 1 contains a collection of covariates considered to confound the association between error-related brain activity and anxiety: sex, racial and ethnic minority status, BI, and anxiety at age 13 years (ie, baseline). The remaining blocks included only neural measures: block 2 contains EEG only, block 3 contains fMRI only, and block 4 contains EEG-fMRI fusion. In light of past work showing that BI substantially moderates the association between error-related brain activity and anxiety,^45^ the corresponding 2-way interactions between brain measures and BI were also included in each block. Additionally, to assess and understand the specific associations that are core to the research questions, we fit a separate path model, where the blocks of neural measures with statistically significant increases in *R*^2^ were tested explaining anxiety change from age 13 to 15 years (Figure 3B).

All statistical analyses were performed with R, version 4.4.1 (R Project for Statistical Computing). SEM models in R were fit with the lavaan package, version 0.6-14,^46^ and significant interactions were probed with the semTools package, version 0.5-6.^47^ All models used full information maximum likelihood estimation, allowing participants with incomplete data to be retained in analyses and providing unbiased estimates even with large proportions of missing data.^48^ Comparisons of baseline measures (collected at or near the time of infant enrollment) between the analytical sample and the rest of the enrolled sample (which was missing all flanker neuroimaging data) are presented in the eTable in Supplement 1. Visualizations of missing data patterns are presented in eFigure 4 in Supplement 1. Two-sided *P* < .05 indicated statistical significance.

## Results

Analyses included 176 participants (84 males at birth [47.7%], 92 females at birth [52.3%]) with any flanker neuroimaging data at age 13 years and 15 years. Of these participants, 5 (2.8%) were identified as Asian individuals, 21 (11.9%) as Black or African American individuals, 11 (6.3%) as Hispanic or Latino individuals, 133 (75.6%) as White individuals, and 6 (3.4%) as individuals of other race and ethnicity.

Mean latent anxiety change did not significantly differ from 0 (standardized estimate, –0.192; unstandardized estimate, –0.76 [95% CI, –5.39 to 3.88]; *P* = .75) (Figure 2), indicating that, in general, anxiety did not significantly differ from age 13 years to 15 years. However, the variance of latent anxiety change scores was significant (standardized estimate, 0.92; unstandardized estimate, 14.30 [95% CI, 2.16-26.44]; *P* = .02), suggesting that individuals differed in their patterns of change over time: some participants experienced increased anxiety, others experienced decreased anxiety, and still others experienced little or no change in anxiety. Across all 3 neural modalities, LCS models revealed significant age-related increases in both error-related dACC (eg, EEG-fMRI fusion: standardized estimate, 0.37; unstandardized estimate, 0.17 [95% CI, 0.08-0.26]; *P* < .001) and PCC activity (eg, EEG-fMRI fusion: standardized estimate, 0.43; unstandardized estimate, 0.18 [95% CI, 0.07-0.29]; *P* = .001) (eFigure 2 in Supplement 1), with significant between-participant variance (eg, EEG-fMRI fusion, dACC: standardized estimate, 0.35; unstandardized estimate, 0.07 [95% CI, 0.04-0.11]; *P* < .001; PCC: standardized estimate, 0.53; unstandardized estimate, 0.09 [95% CI, 0.05-0.14]; *P* < .001), indicating individual differences in change scores.

The incremental validity model (Figure 3A) revealed that block 1 (sex, racial and ethnic minority status, BI, baseline anxiety) explained the greatest amount of variance in anxiety change scores (*R*^2^ = 0.54; *P* < .001). Neither block 2 (EEG only; change in *R*^2^ = 0.01; *P* = .39) nor block 3 (fMRI only; change in *R*^2^ = 0.01; *P* = .65) explained a statistically significant amount of additional variance in anxiety change scores. However, block 4 (EEG-fMRI fusion) did explain a statistically significant amount of additional variance (change in *R*^2^ = 0.25; *P* = .001), beyond the contributions of baseline covariates, EEG-only measures, and fMRI-only measures.

**Figure 4.**
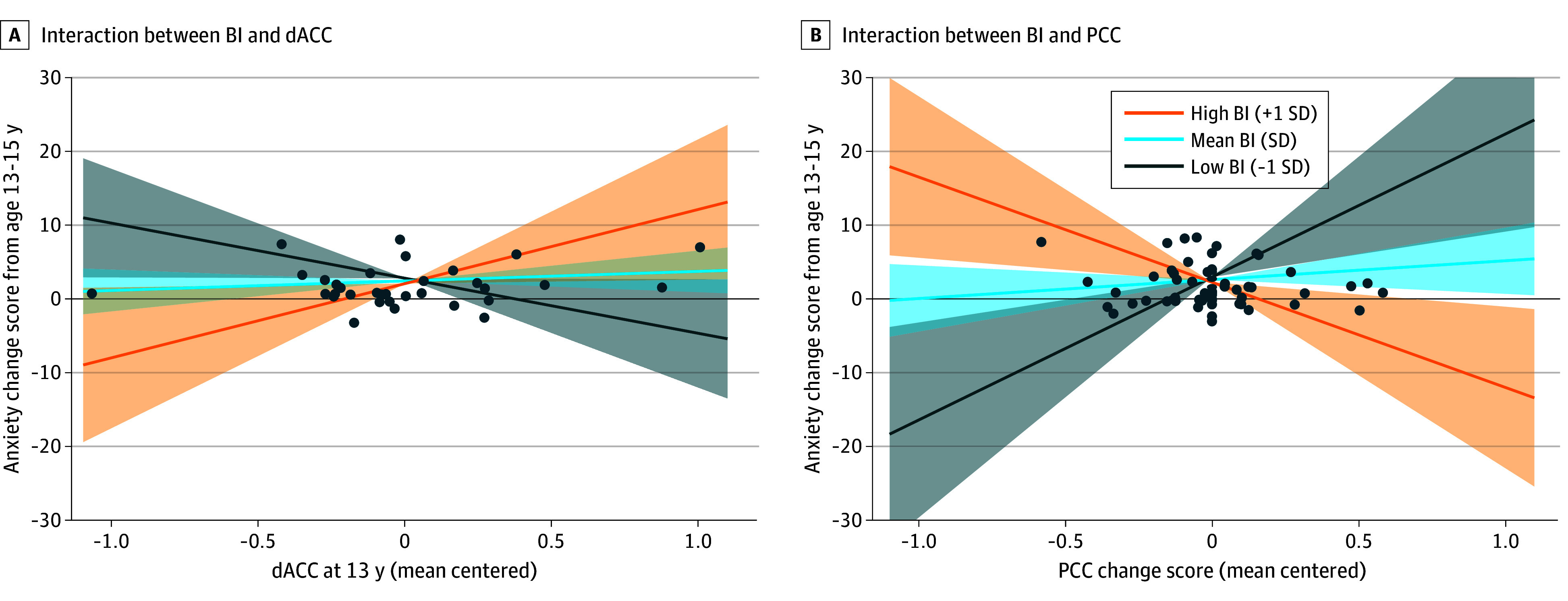
Interactions From Electroencephalogram–Functional Magnetic Resonance Imaging (EEG-fMRI) Fusion Anxiety Estimation Model Although all participants with any flanker neuroimaging data (n = 176) were included in this analysis, plotted data points only include participants with complete data for illustrative purposes. Shading indicates 95% CI. BI indicates behavioral inhibition; dACC, dorsal anterior cingulate cortex; and PCC, posterior cingulate cortex.

Because only the EEG-fMRI fusion block explained significant additional variance in anxiety change scores (change in *R*^2^ = 0.25; *P* = .001), EEG-fMRI fusion measures and their interactions with temperament were included in a new path model estimating anxiety change (Figure 3B). Factors in this path model included sex, racial and ethnic minority status, BI, baseline anxiety as well as baseline dACC and PCC activity scores, change in dACC and PCC activity scores, and their interactions with BI (a path model that additionally controlled for maternal education is shown in eFigure 5 in Supplement 1). Greater age-related increases in dACC activity were associated with greater decreases in anxiety (*β* = –0.41; *B* = −4.90; 95% CI, –8.87 to 0.93; *P* = .02). There was a significant interaction between BI and dACC activity at age 13 years (*β* = 0.40; *B* = 8.77; 95% CI, 0.74-16.79; *P* = .03) (Figure 4A), indicating that the association between 13th-year dACC activity and anxiety change scores significantly differed by BI history. Simple slopes analysis revealed that among youths with high BI, greater 13th-year dACC activation was associated with increased anxiety from age 13 to 15 years (*B* = 10.08; 95% CI, 0.56-19.60; *P* = .04). Among youths with low BI, greater 13th-year dACC activation was instead associated with decreased anxiety from age 13 to 15 years (*B* = −7.49; 95% CI, –14.84 to 0.14; *P* = .046). At mean levels of BI, the association between 13th-year dACC activity and anxiety change was not significant (*B* = 1.29; 95% CI, –1.53 to 4.11; *P* = .37).

This model also revealed a significant interaction between BI and PCC activity change scores (*β* = –0.42; *B* = −16.89; 95% CI, –28.21 to –5.57; *P* = .003) (Figure 4B), indicating that the association between PCC activity change scores and anxiety change scores significantly differed by BI history. Simple slopes analysis indicated that among youths with high BI, greater age-related increases in PCC activity were associated with decreased anxiety over time (*B* = −14.38; 95% CI, –25.35 to 3.42; *P* = .01), whereas the opposite was true among youths with low BI (*B* = 19.54; 95% CI, 6.29-32.78; *P* = .004). At mean levels of BI, the association between PCC activity change scores and anxiety change scores was not significant (*B* = 2.58; 95% CI, –1.92 to 7.07; *P* = .26).

## Discussion

In this cohort study, after covariates explained 54% (*R*^2^ = 0.54) of variance in anxiety change scores, EEG source and fMRI measures each added approximately 1% (*R*^2^ = 0.01) of variance. EEG-fMRI fusion scores explained an additional 25% (*R*^2^ = 0.25) of variance in anxiety change scores (beyond all other factors). The integration of complementary spatial and temporal precision of EEG and fMRI was associated with markedly improved estimation of future anxiety in youth.

A follow-up path model revealed an association between 13th-year dACC activity and anxiety change scores, which varied with BI history: greater dACC activity was associated not only with future anxiety increases among adolescents with a history of high BI but also with future anxiety decreases among those with a history of low BI. This finding aligns with results of prior work in a cohort of adolescents linking larger ERN amplitudes to greater odds of having a concurrent anxiety diagnosis, but only among adolescents with a history of high BI.^16^ The present results add further nuance and indicate that ERN-related activity in the dACC, specifically, may explain the associations among BI, the ERN, and anxiety while also enabling estimation of future anxiety functioning.

Although ERN-related activity in the PCC at age 13 years was not associated with anxiety changes across time, changes in PCC activity from age 13 to 15 years were associated with changes in anxiety over the same period. This association also varied as a function of early BI history; among adolescents with high BI, increases in PCC activity were associated with decreases in anxiety, whereas among youths with low BI the opposite pattern emerged. Developmental work shows the ERN shifts from being primarily dACC-driven in preadolescence to more PCC-driven across adolescence and into adulthood,^24^ possibly indicating increased attentional flexibility with age.^26^ Considering this developmental finding, the present results suggest that, for adolescents with a history of BI, having a less mature pattern of error monitoring activity (characterized by heightened dACC activation at age 13 years) may increase future anxiety risk. In contrast, undergoing greater maturational changes in error monitoring activity (characterized by larger increases in PCC activation over time) may help alleviate this risk. For adolescents without a history of high BI, however, the opposite pattern emerged, highlighting the importance of temperamental differences in differentiating risk trajectories.

Only 1 association involving EEG-fMRI fusion scores was not qualified by temperament history. Across the whole sample, age-related increases in dACC activity from age 13 to 15 years were associated with decreased anxiety across time. Although this study, to our knowledge, is among the first longitudinal research of its kind, cross-sectional work has found that the overall association between ERN amplitude and anxiety differs across development.^20,26,27,28^ A previous review noted that larger ERNs were associated with elevated anxiety among older adolescents, but for younger children anxiety was associated with smaller ERNs.^20^ Yet, the same review also found that larger ERNs were associated with clinically significant anxiety for both younger children and older adolescents. This paradoxical pattern may be partly explained by our finding of distinct associations between ERN-related activity and anxiety across the dACC and PCC—2 brain regions whose unique contributions to the ERN change across development. Overall, because effect sizes of EEG-fMRI fusion scores and their interactions were in the medium range, the findings are likely clinically relevant,^49^ suggesting that these neurobiological markers may be useful in identifying adolescents at risk for anxiety.

### Limitations

Although this study had numerous strengths, including the use of longitudinal data, multimethod assessment of anxiety, and novel EEG-fMRI fusion approach, it had several notable limitations. First, crossover interactions were observed between BI and neural measures. Crossover interactions should be interpreted with caution,^50^ particularly when the 2 interacting variables are highly correlated and the sample size is small. In our study, BI was not associated with error responses (and age-related changes thereof) and the sample size was moderate, suggesting that findings may not be attributed to discordant cells of small sample size. Nevertheless, caution is warranted in interpreting these interactions. Second, participants from racial and ethnic minority groups were more likely to have missing adolescent neuroimaging data, which may limit generalizability. Lastly, of all variables involved in analyses, EEG-fMRI fusion scores were the most likely to be missing because their computation required both EEG and fMRI data. However, this lack of data was mitigated in all analyses by the use of full information maximum likelihood estimation.

## Conclusions

In this cohort study, integrating EEG and fMRI measures of error monitoring significantly improved estimations of changes in adolescents’ future anxiety. EEG-fMRI fusion scores explained additional variance in anxiety change scores over and above demographics, temperament history, baseline anxiety scores, and measures derived from each neuroimaging modality separately. History of BI temperament interacted with error monitoring scores to differentiate anxiety trajectories, but these interactions differed by brain region, highlighting the value of harnessing the complementary spatial and temporal resolution of EEG and fMRI to increase measurement precision.

## Notes

Supplement 1.
eMethods.
**eTable.** Missing Data Comparisons **eFigure 1.** Pearson Correlations Across Key Study Variables **eFigure 2.** Latent Change Score Models for Neural Measures of Error Monitoring **eFigure 3.** Complete Incremental Predictive Validity Model **eFigure 4.** Missing Data Patterns **eFigure 5.** EEG-fMRI Fusion Anxiety Prediction Model Controlling for Highest Level of Maternal
eReferences.

Supplement 2.
Data Sharing Statement
