# Error in Figure 1

**DOI:** 10.1001/jamanetworkopen.2025.49498

**Published:** 2025-11-21

In the Original Investigation titled “Integrating Multimodal Neuroimaging of Error Monitoring to Estimate Future Anxiety in Adolescents”^1^ published October 23, 2025, there was an error in Figure 1. The color bar under the functional magnetic resonance images was incorrect. This article has been corrected.^1^
