# Supplementary material for: Integrating Multimodal Neuroimaging of Error Monitoring to Estimate Future Anxiety in Adolescents
Source: JAMA Netw Open. 2025 Oct 23;8(10):e2539133. doi: 10.1001/jamanetworkopen.2025.39133 (PMC12550640; doi:10.1001/jamanetworkopen.2025.39133)
Supplement: Supplement 1. — eMethods. eTable. Missing Data Comparisons eFigure 1. Pearson Correlations Across Key Study Variables eFigure 2. Latent Change Score Models for Neural Measures of Error Monitoring eFigure 3. Complete Incremental Predictive Validity Model eFigure 4. Missing Data Patterns eFigure 5. EEG-fMRI Fusion Anxiety Prediction Model Controlling for Highest Level of Maternal eReferences. [file jamanetwopen-e2539133-s001.pdf]

Supplemental Online Content

Valadez EA, Conte S, Richards JE, et al. Integrating multimodal neuroimaging of error monitoring to estimate future anxiety in adolescents. *JAMA Netw Open*. 2025;8(10):e2539133. doi:10.1001/jamanetworkopen.2025.39133

eMethods.

eTable. Missing Data Comparisons

eFigure 1. Pearson Correlations Across Key Study Variables

eFigure 2. Latent Change Score Models for Neural Measures of Error Monitoring

eFigure 3. Complete Incremental Predictive Validity Model

eFigure 4. Missing Data Patterns

eFigure 5. EEG-fMRI Fusion Anxiety Prediction Model Controlling for Highest Level of Maternal

eReferences.

This supplemental material has been provided by the authors to give readers additional information about their work.

© 2025 Valadez EA et al. *JAMA Network Open*

eMethods.

Behavioral Inhibition

BI was assessed at ages 24 and 36 months via behavioral coding of laboratory assessments [40,41]. During these laboratory assessments, children were presented with an unfamiliar adult and various novel toys across three sessions at each age (total six sessions). Measures of interest included physical proximity to mother and latency to vocalize, to approach and touch the toys, and to approach the stranger. Each observational measure was standardized and converted to a Z-score. An individual's Z-scores from all measures and sessions were averaged to create a single composite BI score. Contrary to some past approaches which also incorporated parental reports of toddlers' social fear (e.g., [10]), the present BI composite included only observational measures to avoid shared reporter variance with the anxiety outcome, which relied in part on parental reports

Screen for Child Anxiety Related Emotional Disorders

At ages 13 and 15 years, participants and their parent separately reported on the participant's anxiety symptoms via the revised version of the Screen for Child Anxiety Related Emotional Disorders (SCARED)[1]. The parent and child versions of SCARED included 41 items presented on a 3-point Likert scale (0 = never/hardly ever true, 1 = sometimes/somewhat true, 2 = very/often true). Total SCARED scores at each time point and from each reporter were included as indicators in latent change score analyses. Internal consistency (Cronbach's alpha and McDonald's omega) was excellent at both assessments and for both reporters (13-year parent: $\alpha = 0.92$, $\omega = .94$; 13-year child: $\alpha = 0.92$, $\omega = .93$; 15-year parent: $\alpha = 0.93$, $\omega = .94$; 15-year child: $\alpha = 0.93$, $\omega = .94$).

Schedule for Affective Disorders and Schizophrenia for School-Age Children

Participants were administered the Schedule for Affective Disorders and Schizophrenia for School-Age Children–Present and Lifetime version (K-SADS-PL)[2] at both ages 13 and 15

years. K-SADS-PL is a semistructured diagnostic interview for assessing current and past psychopathology among children and adolescents according to *DSM-5* criteria. It was administered by an advanced graduate student or doctoral-level clinician, under the close supervision of a board-certified child and adolescent psychiatrist and a licensed clinical psychologist. At both time points, reliability across raters was acceptable (K > .8)[3,4]. The presence or absence of at least one anxiety disorder, coded as a binary score at each time point, was included as an indicator in latent change score analyses (see Data Analytic Strategy for details). Of the 131 participants administered K-SADS-PL at age 13 years, 18 (13.7%) currently met criteria for at least one anxiety disorder. Of the 151 participants interviewed at age 15 years, 46 (30.5%) met criteria for at least one anxiety disorder. Participants without K-SADS-PL data were included in analyses if they had at least one other measure of interest (see Statistical Analysis section of Methods).

Flanker Task

Each trial of the flanker task involved presentation of a central arrow flanked by two additional arrows on each side. The flanker arrows faced either the same direction as the central arrow (congruent trials; e.g., >>>>>) or the opposite direction (incongruent trials; e.g., <<><<). Participants were instructed to indicate the direction of the central arrow via a button press, ignoring the flanker arrows. Incongruent and congruent trials were presented with equal probability. Each trial began with a 300–600 ms fixation cross; next, the flanker stimulus appeared for 200 ms, followed by either an 1860-ms (in the EEG session) or 1700-ms (in the fMRI session) blank screen. The EEG session consisted of 10 blocks of 32 trials (total 320 trials). The fMRI session consisted of 4 six-minute runs, each comprised of 108 trials (total 432 trials). At the end of each block, computer-generated feedback was presented to the participant to help maintain accuracy at a level ensuring enough errors for analysis.

EEG Acquisition and Processing

EEG was recorded using a 128-channel Hydrocel Geodesic Sensor Net and EGI software (Electrical Geodesic, Inc., Eugene, OR). Data were sampled at 250 Hz with electrode impedance maintained below 50kΩ. Offline processing was performed with the Maryland Analysis of Developmental EEG (MADE) pipeline [5] (v1.0). The MADE pipeline is implemented in MATLAB (The MathWorks, Natick, MA) and uses the toolbox EEGLAB[6] and its plugins "firfilt", FASTER [7], ADJUST [8], and Adjusted-ADJUST[9]. All default options of the MADE pipeline were used. Data were re-referenced to an average reference and filtered with a Hamming windowed digital FIR filter. Data were high pass filtered at 0.3 Hz with a half-amplitude cutoff frequency of 0.15 Hz. Data were low pass filtered at 50 Hz with a half-amplitude cutoff frequency of 55 Hz. FASTER was used to identify globally bad channels [7]. Additionally, channels were marked bad at the epoch level if voltage exceeded ±125 µV within the epoch, and any epochs in which more than 10% of non-ocular channels exceeded this threshold were marked bad. Otherwise, bad channels were interpolated via a spherical-spline interpolation. Ocular artifact detection and removal was performed with ICA (see [5] for detailed ICA steps) paired with Adjusted-ADJUST [9], an automated algorithm for identifying artifactual ICA components.

To facilitate source localization, the 3D coordinates of 14 fiducial landmarks were digitized during the EEG session using Polhemus Fastrak. Using these coordinates, electrode positions were co-registered with the structural MRI and interpolated to form standardized virtual electrodes in 10-10 space [10,11].

Epochs were segmented from -100 to 500 ms relative to response onset. Baseline adjustment was performed by subtracting single-trial voltage values by that trial's mean voltage during the 50-ms period preceding response onset. This smaller baseline period was used to minimize contamination from earlier ERP components. Incongruent error trials and incongruent correct trials were averaged separately across a cluster of 10-10 electrodes including AFz, Fz,

FCz, Cz, and CPz. The ΔERN was quantified as the peak negative voltage difference between incongruent error and incongruent correct trials within the window of 0–100 ms post-response.

MRI Acquisition

T1- and T2-weighted whole-brain structural MRI images were acquired in a 3-Tesla MR750 GE scanner with a 32-channel head coil. The T1-weighted scan was a magnetization-prepared rapid acquisition gradient-echo sequence (MPRAGE; sagittal acquisition; TI/TE = 425/min; flip angle = 7°; FOV = 25.6; Matrix 256 × 256; Slice thickness = 1 mm; bandwidth = 25 Hz). The T2-weighted scan was a fast relaxation fast spin-echo sequence was acquired (FRFSE-XL; sagittal acquisition; TR/TE 15000/80 ms; FOV = 25.6; Matrix 256 × 256; Slice thickness = 1 mm; bandwidth = 31.25 Hz). Functional MRI images were obtained during the flanker task and consisted of 170 whole-brain T2-weighted echoplanar images (TR = 2000 ms, TE = 25, flip angle, 60 deg, 24 field of view, 96 x 96 matrix).

At each time point, age-appropriate average brain templates were selected from the Neurodevelopmental MRI Database [12]. The templates were used in the electrode co-registration procedure, to display group-level results of source data, for fMRI display and analysis, and for relating MNI templates to ages and individuals. Each participant's structural MRI was registered to the age-appropriate average template using FSL's "flirt" function [13,14]. The fMRI volumes were registered to the T2-weighted volume by placing the images from the four flanker task runs into a single 4D volume, using FSL's MCFLIRT [14] to do motion correction, obtaining the mean BOLD volume from the motion corrected images, and using FSL flirt [13,14] to register to the T2-weighted MRI volume.

ERN Source Analysis

Cortical source analysis was used to identify the cortical generators of the ERN. Steps applied to each participant's data included: 1) segmenting the head using T1- and T2-weighted scans into component media with varying conductivity values; 2) computing a volume

conduction model (i.e., head model) describing how electrical current flows through the head, represented as a tetrahedral mesh; 3) creating a source model consisting of segmented gray matter and eyes, also as a tetrahedral mesh; 4) identifying the electrode positions on the T1-weighted MRI; 5) computing a lead-field matrix mapping the relations between the 128 electrodes and the voxels in the source model; 6) computing the inverse spatial filter created with the exact low-resolution electromagnetic tomography (eLORETA) method [15], which is then multiplied by the participant's scalp EEG data to reconstruct the signal's current density. This final step results in a sample-by-sample estimate of current density reconstructed (CDR) values for each voxel in the cortex. Source analysis was performed separately for incongruent-error and incongruent-correct trials. ΔCDR values were obtained by subtracting incongruent-correct CDR from incongruent-error CDR (Figure 1a, left).

For subsequent structural equation models, data were reduced by first calculating the mean ΔCDR level at each sample across two a priori ROIs (dACC and PCC; Figure 1b) and then convolving the ΔCDR time course with the peaked ΔERN using a quadratic polynomial filter. The anatomical ROIs were defined for each individual participant's brain using a combination of atlases in subject-specific MRI space. For detailed description of methods used to create the anatomical ROIs, see [16].

fMRI Analysis

The fMRI preprocessing pipeline was designed by Hanayik and Richards[17] and uses functions from the FSL (version 5.0) [18] and SPM 12 [19] software packages. Key steps included head motion detection with FSL's MCFLIRT, spatial smoothing with FSL's fslmaths, and a general linear model (GLM) with an event-related design implemented in SPM 12. Each of the four fMRI task runs was processed separately. The GLM used a temporal derivative for the hemodynamic response function with regressors including the onset times for each trial, classified as congruent-correct, incongruent-correct, and incongruent-error (congruent-error was

omitted due to too few trials and because there were no hypotheses concerning this trial type). Analyses focused on an incongruent-error > incongruent-correct contrast; values from this contrast were concatenated into a single 4D MRI volume and tested as a one-way analysis in PALM[20]. The BOLD fMRI volumes were concatenated, summed, then registered to the T2-weighted MRI volumes with FSL flirt to put them in the same space as the CDR results (Figure 1a, middle).

eTable. *Missing Data Comparisons*

Variable	Included in analyses (n = 176) *M/n*	*SD/%*	Excluded from analyses (n = 115) *M/n*	*SD/%*	Test statistic	*p*-value
Child sex at birth (male)	84	47.7%	51	44.3%	$X^2(1, N=291) = 0.20$	.66
Racial/ethnic minority status	43	24.4%	45	39.5%	$X^2(1, N=290) = 6.71$	.01
Highest maternal education					$X^2(2, N=273) = 0.19$	.99
High school diploma	29	17.5%	18	16.8%		
College degree	74	44.6%	48	45.0%		
Postgraduate degree	63	38.0%	41	38.3%		
Behavioral inhibition	-0.02	0.44	0.03	0.51	$t(246) = 0.76$	.45
Infant positive reactivity	17.08	16.44	19.74	23.51	$t(289) = 1.14$	.26
Infant negative reactivity	23.75	25.61	24.46	30.54	$t(289) = 0.21$	.83
Infant motor reactivity	29.62	17.52	29.91	20.52	$t(289) = 0.13$	.90

Variable	Infants followed longitudinally (n = 291) *M*	*SD*	Unselected infants (n = 488) *M*	*SD*	Test statistic	*p*-value
Infant positive reactivity	18.13	19.55	14.66	17.05	$t(777) = -2.60$	.01
Infant negative reactivity	24.03	27.61	17.39	25.62	$t(777) = -3.40$	< .001
Infant motor reactivity	29.74	18.72	10.06	11.41	$t(776) = -18.22$	< .001

Note: Participants were included in analyses if they had data from at least one of the adolescent neuroimaging assessments (n = 176). All variables in table were assessed at or near the time of infant enrollment. Due to small cell sizes, racial/ethnic minoritized status was coded as Non-Hispanic White = 0, all others = 1.

© 2025 Valadez EA et al. *JAMA Network Open*

eFigure 1. *Pearson Correlations Across Key Study Variables*

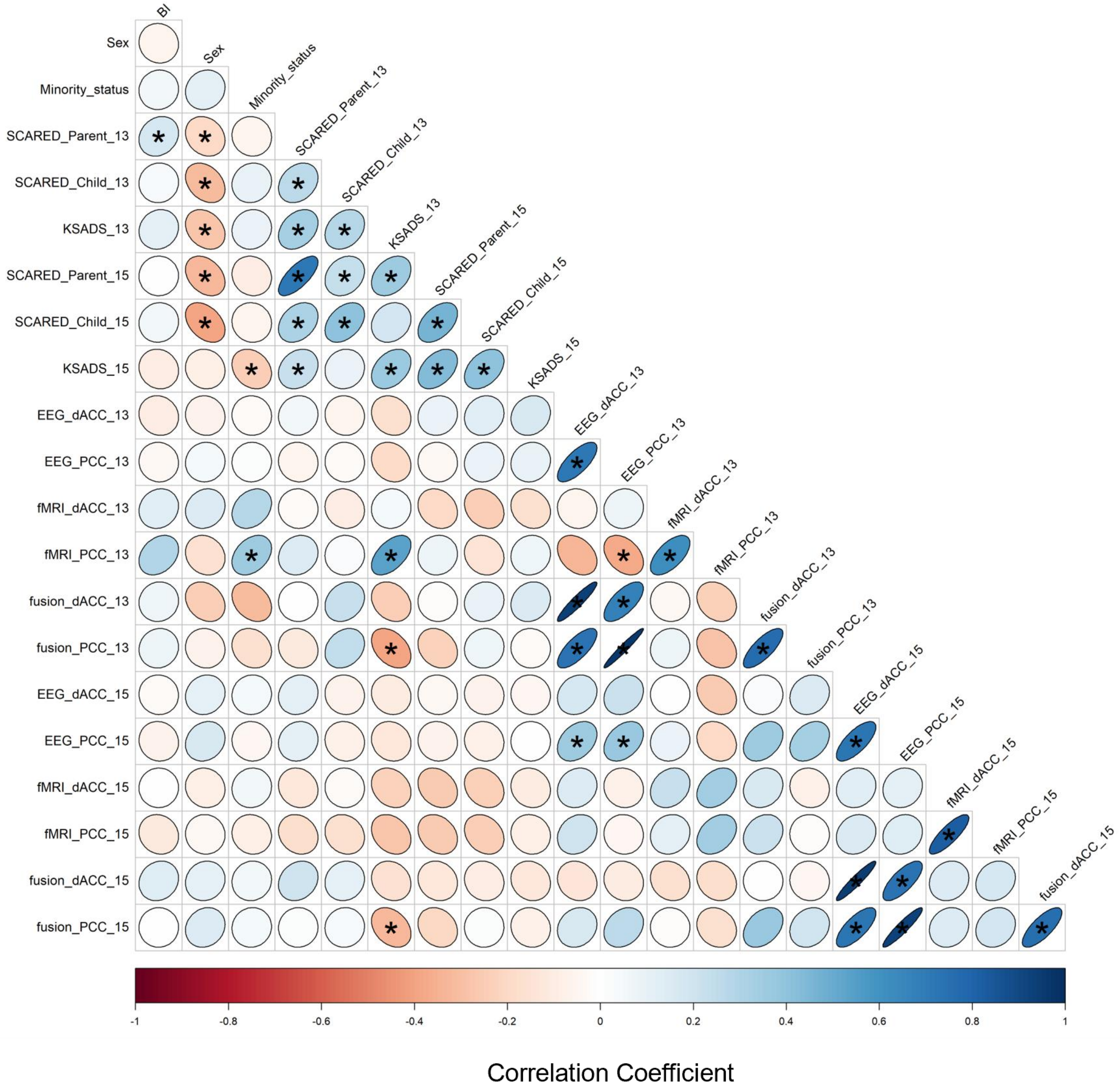

Note: Numbers in variable names indicate time of assessment (e.g., 13 = age 13 years, 15 = age 15 years). SCARED = Screen for Child Anxiety Related Emotional Disorders. KSADS = Schedule for Affective Disorders and Schizophrenia for School-Age Children. dACC = dorsal anterior cingulate cortex. PCC = posterior cingulate cortex. *$p < .05$.

© 2025 Valadez EA et al. *JAMA Network Open*

eFigure 2. *Latent Change Score Models for Neural Measures of Error Monitoring*

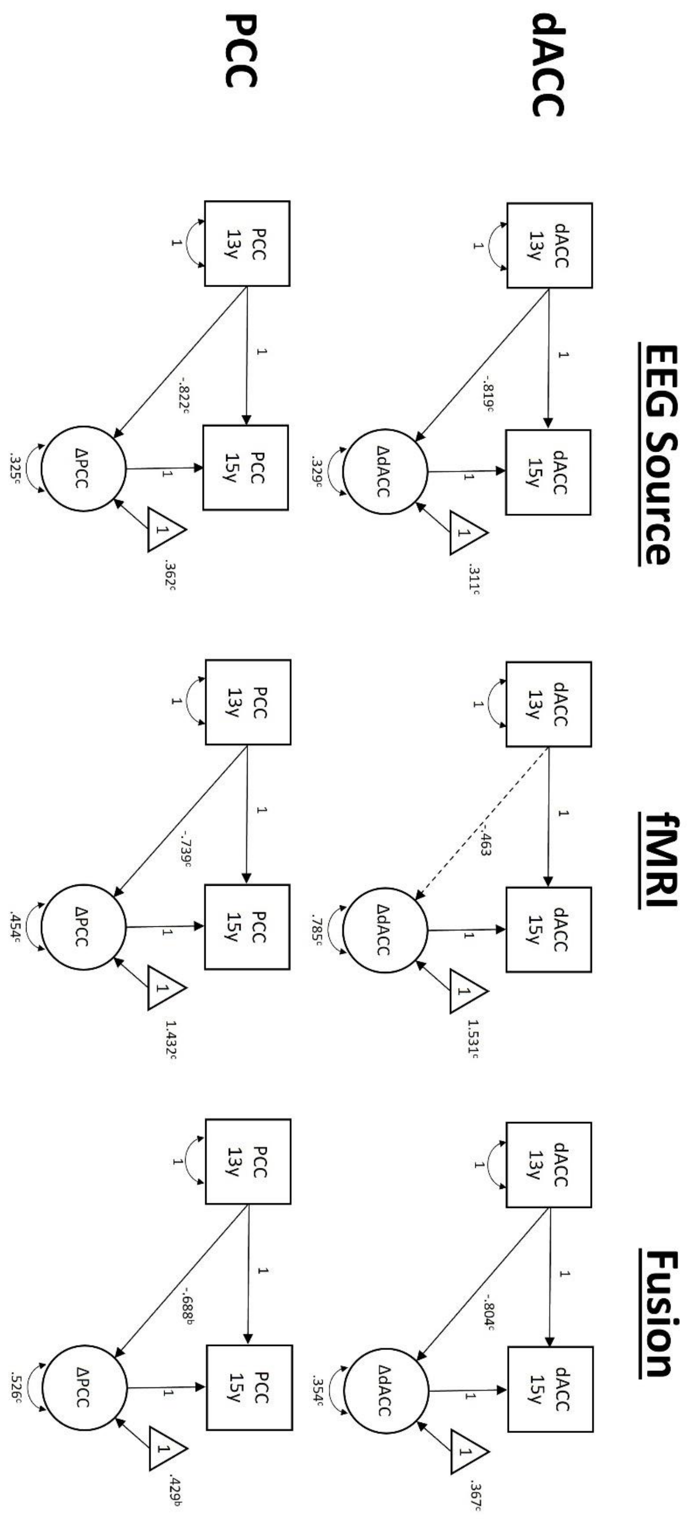

© 2025 Valadez EA et al. *JAMA Network Open*

Note: Standardized estimates are shown. Fit indices are not reported because univariate, single indicator latent change score models are identified. dACC = dorsal anterior cingulate cortex. PCC = posterior cingulate cortex. [a]$p < .05$; [b]$p < .01$; [c]$p < .001$.

© 2025 Valadez EA et al. *JAMA Network Open*

eFigure 3. *Complete Incremental Predictive Validity Model*

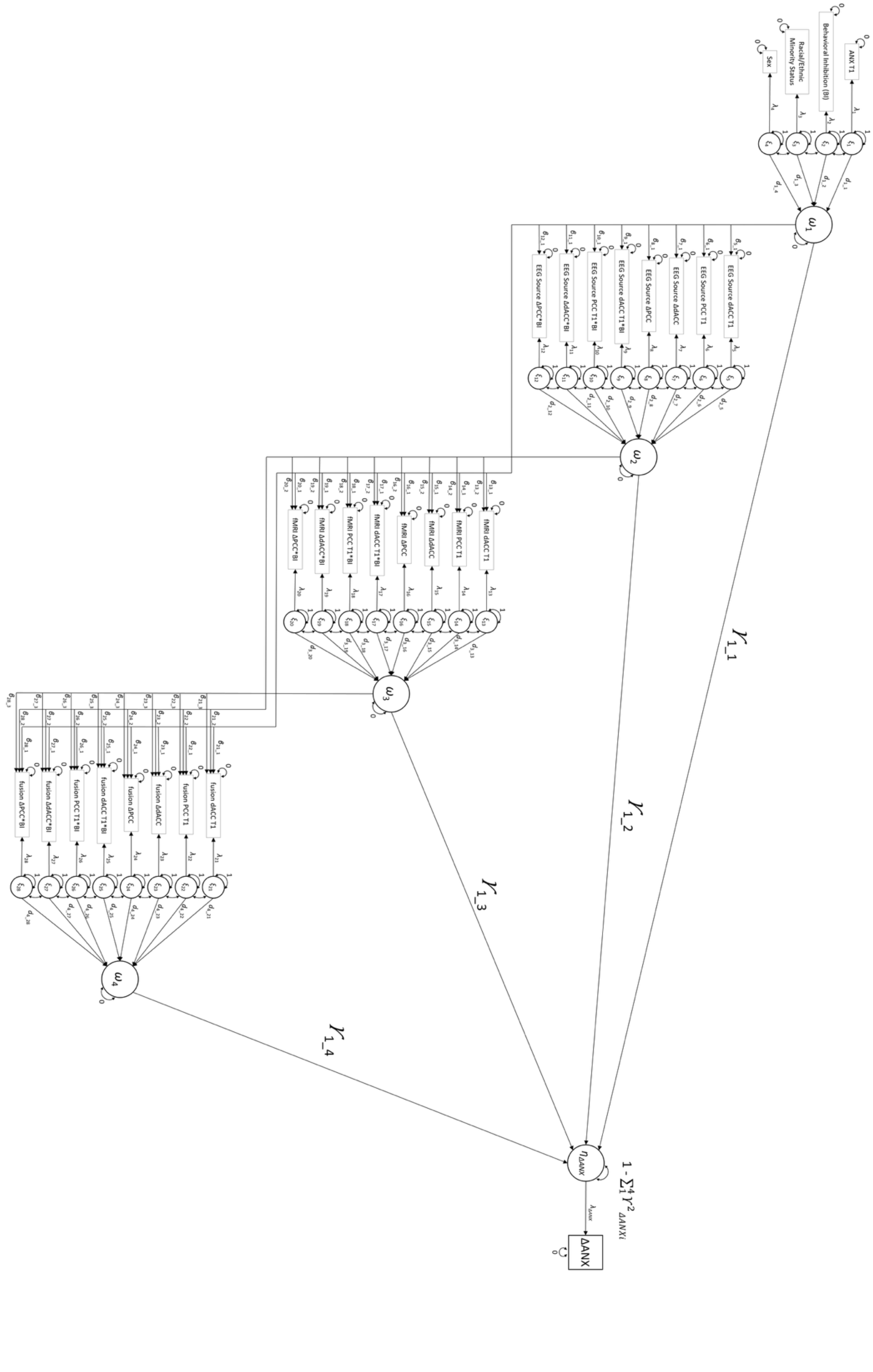

© 2025 Valadez EA et al. *JAMA Network Open*

Note: For clarity, not all covariances among phantom latent variables are shown. Specifically, ξ1-4 all covary with one another, as do ξ5-12, ξ13-20, and ξ21-28. ANX = Anxiety. dACC = dorsal anterior cingulate cortex. PCC = posterior cingulate cortex. BI = behavioral inhibition.

© 2025 Valadez EA et al. *JAMA Network Open*

eFigure 4. *Missing Data Patterns*

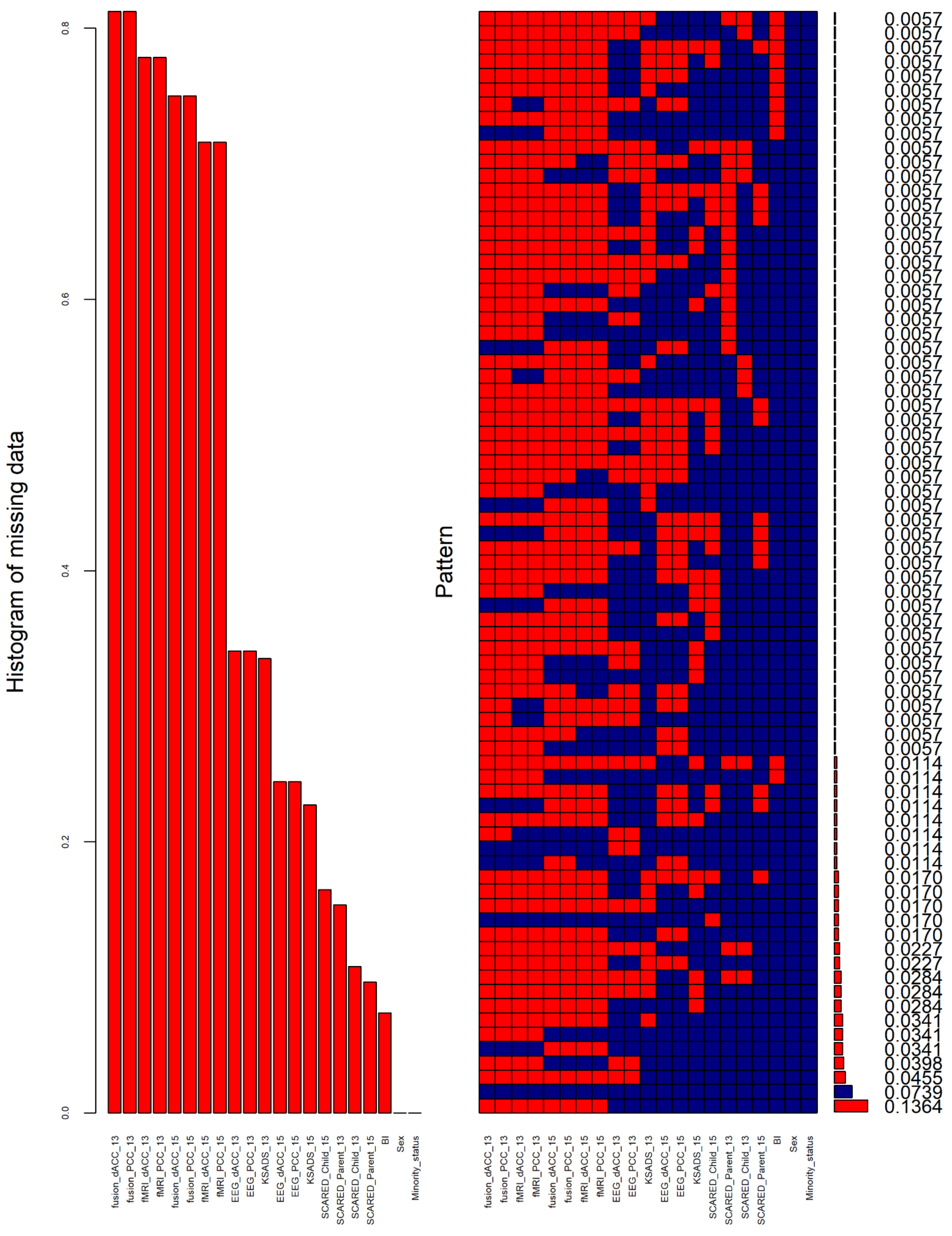

© 2025 Valadez EA et al. *JAMA Network Open*

Note: Missing data shown in red. SCARED = Screen for Child Anxiety Related Emotional Disorders. KSADS = Schedule for Affective Disorders and Schizophrenia for School-Age Children. dACC = dorsal anterior cingulate cortex. PCC = posterior cingulate cortex.

© 2025 Valadez EA et al. *JAMA Network Open*

eFigure 5. *EEG-fMRI Fusion Anxiety Prediction Model Controlling for Highest Level of Maternal Education*

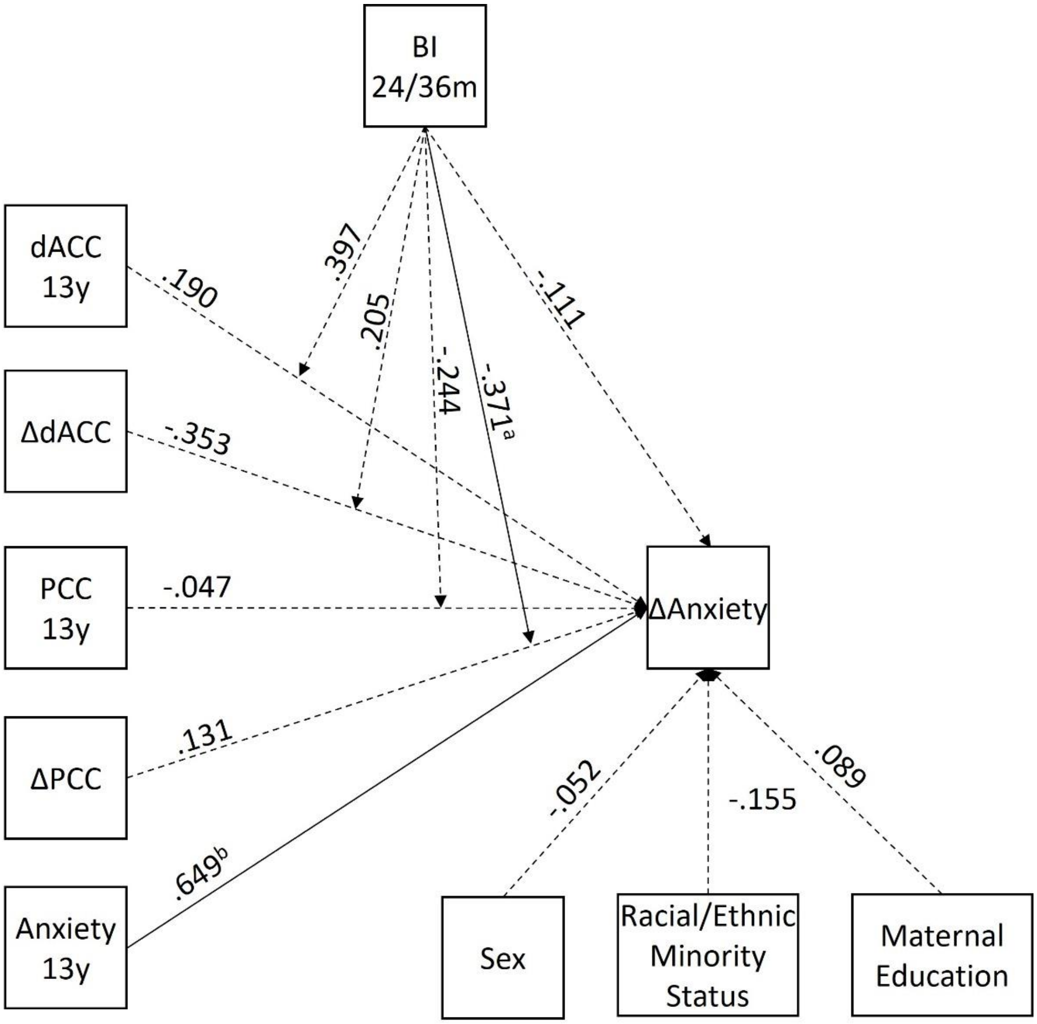

Note: Upon enrollment in the longitudinal study, participants' mothers reported on their highest level of education. Maternal education was coded on an ordinal scale such that high school diploma = 0, college degree = 1, and postgraduate degree = 2. Therefore, higher scores indicate greater educational attainment. Path coefficients are standardized. Fit indices are not reported because models are just-identified. ANX = anxiety. dACC = dorsal anterior cingulate cortex. PCC = posterior cingulate cortex. BI = behavioral inhibition. [a]*p* < .05; [b]*p* < .01; [c]*p* < .001.

© 2025 Valadez EA et al. *JAMA Network Open*

eReferences

1. Birmaher B, Khetarpal S, Brent D, et al. The Screen for Child Anxiety Related Emotional Disorders (SCARED): Scale Construction and Psychometric Characteristics. *J Am Acad Child Adolesc Psychiatry*. 1997;36(4):545-553. doi:10.1097/00004583-199704000-00018

2. Kaufman J, Birmaher B, Brent D, et al. Schedule for Affective Disorders and Schizophrenia for School-Age Children-Present and Lifetime Version (K-SADS-PL): Initial Reliability and Validity Data. *J Am Acad Child Adolesc Psychiatry*. 1997;36(7):980-988. doi:10.1097/00004583-199707000-00021

3. Buzzell GA, Troller-Renfree SV, Barker TV, et al. A neurobehavioral mechanism linking behaviorally inhibited temperament and later adolescent social anxiety. *J Am Acad Child Adolesc Psychiatry*. 2017;56(12):1097-1105. doi:10.1016/j.jaac.2017.10.007

4. Morales S, Zeytinoglu S, Lorenzo NE, et al. Which Anxious Adolescents Were Most Affected by the COVID-19 Pandemic? *Clin Psychol Sci*. 2022;10(6):1044-1059. doi:10.1177/21677026211059524

5. Debnath R, Buzzell GA, Morales S, Bowers ME, Leach SC, Fox NA. The Maryland analysis of developmental EEG (MADE) pipeline. *Psychophysiology*. 2020;57(6):e13580. doi:10.1111/psyp.13580

6. Delorme A, Makeig S. EEGLAB: An open source toolbox for analysis of single-trial EEG dynamics including independent component analysis. *J Neurosci Methods*. 2004;134(1):9-21. doi:10.1016/j.jneumeth.2003.10.009

7. Nolan H, Whelan R, Reilly RB. FASTER: Fully automated statistical thresholding for EEG artifact rejection. *J Neurosci Methods*. 2010;192(1):152-162. doi:10.1016/j.jneumeth.2010.07.015

8. Mognon A, Jovicich J, Bruzzone L, Buiatti M. ADJUST: An automatic EEG artifact detector based on the joint use of spatial and temporal features. *Psychophysiology*. 2011;48(2):229-240. doi:10.1111/j.1469-8986.2010.01061.x

9. Leach SC, Morales S, Bowers ME, et al. Adjusting ADJUST: Optimizing the ADJUST algorithm for pediatric data using geodesic nets. *Psychophysiology*. 2020;57(8):e13566.

10. Conte S, Richards JE. Cortical Source Analysis of Event-Related Potentials: A Developmental Approach. *Dev Cogn Neurosci*. 2022;54:101092. doi:10.1016/j.dcn.2022.101092

© 2025 Valadez EA et al. *JAMA Network Open*

11. Jurcak V, Tsuzuki D, Dan I. 10/20, 10/10, and 10/5 systems revisited: Their validity as relative head-surface-based positioning systems. *NeuroImage*. 2007;34(4):1600-1611. doi:10.1016/j.neuroimage.2006.09.024

12. Richards JE, Xie W. Chapter One - Brains for All the Ages: Structural Neurodevelopment in Infants and Children from a Life-Span Perspective. In: Benson JB, ed. *Advances in Child Development and Behavior*. Vol 48. JAI; 2015:1-52. doi:10.1016/bs.acdb.2014.11.001

13. Jenkinson M, Smith S. A global optimisation method for robust affine registration of brain images. *Med Image Anal*. 2001;5(2):143-156. doi:10.1016/S1361-8415(01)00036-6

14. Jenkinson M, Bannister P, Brady M, Smith S. Improved Optimization for the Robust and Accurate Linear Registration and Motion Correction of Brain Images. *NeuroImage*. 2002;17(2):825-841. doi:10.1006/nimg.2002.1132

15. Pascual-Marqui RD, Lehmann D, Koukkou M, et al. Assessing interactions in the brain with exact low-resolution electromagnetic tomography. *Philos Trans R Soc Math Phys Eng Sci*. 2011;369(1952):3768-3784. doi:10.1098/rsta.2011.0081

16. Buzzell GA, Richards JE, White LK, Barker TV, Pine DS, Fox NA. Development of the error-monitoring system from ages 9–35: Unique insight provided by MRI-constrained source localization of EEG. *NeuroImage*. 2017;157:13-26. doi:10.1016/j.neuroimage.2017.05.045

17. Hanayik T, Richards J. *Preprocessing and Processing Pipeline for fMRI*.; 2018. doi:10.13140/RG.2.2.36556.46722

18. Smith SM, Jenkinson M, Woolrich MW, et al. Advances in functional and structural MR image analysis and implementation as FSL. *NeuroImage*. 2004;23:S208-S219. doi:10.1016/j.neuroimage.2004.07.051

19. Penny WD, Friston KJ, Ashburner JT, Kiebel SJ, Nichols TE. *Statistical Parametric Mapping: The Analysis of Functional Brain Images*. Elsevier; 2011.

20. Winkler AM, Ridgway GR, Webster MA, Smith SM, Nichols TE. Permutation inference for the general linear model. *NeuroImage*. 2014;92:381-397. doi:10.1016/j.neuroimage.2014.01.060

© 2025 Valadez EA et al. *JAMA Network Open*
