# Supplementary material for: Integrating Multimodal Neuroimaging of Error Monitoring to Estimate Future Anxiety in Adolescents
Source: JAMA Netw Open. 2025 Oct 23;8(10):e2539133. doi: 10.1001/jamanetworkopen.2025.39133 (PMC12550640; doi:10.1001/jamanetworkopen.2025.39133)
Supplement: Supplement 2. — Data Sharing Statement [file jamanetwopen-e2539133-s002.pdf]

Data Sharing Statement

Valadez. Integrating Multimodal Neuroimaging of Error Monitoring to Estimate Future Anxiety in Adolescents. *JAMA Netw Open*. Published October 23, 2025.
doi:10.1001/jamanetworkopen.2025.39133

Data
Data available: Yes
Data types: Deidentified participant data
How to access data: https://nda.nih.gov/edit_collection.html?id=2538
When available: With publication

Supporting Documents
Document types: None

Additional Information
Who can access the data: Researchers whose proposed use of the data has been approved
Types of analyses: For any research purpose
Mechanisms of data availability: With an approved Data Use Agreement (DUC; see https://nda.nih.gov for details)
